# Increased expression of programmed death ligand 1 (PD-L1) in human pituitary tumors

**DOI:** 10.18632/oncotarget.12088

**Published:** 2016-09-17

**Authors:** Yu Mei, Wenya Linda Bi, Noah F. Greenwald, Ziming Du, Nathalie Y. R. Agar, Ursula B. Kaiser, Whitney W. Woodmansee, David A. Reardon, Gordon J. Freeman, Peter E. Fecci, Edward R. Laws, Sandro Santagata, Gavin P. Dunn, Ian F. Dunn

**Affiliations:** ^1^ Department of Neurosurgery, Brigham and Women's Hospital, Harvard Medical School, Boston, MA, USA; ^2^ Department of Cancer Biology, Dana-Farber Cancer Institute, Harvard Medical School, Boston, MA, USA; ^3^ Department of Pathology, Brigham and Women's Hospital, Harvard Medical School, Boston, MA, USA; ^4^ Division of Endocrinology, Brigham and Women's Hospital, Harvard Medical School, Boston, MA, USA; ^5^ Department of Medical Oncology, Dana-Farber Cancer Institute, Harvard Medical School, Boston, MA, USA; ^6^ Department of Neurosurgery, Duke University School of Medicine, Durham, NC, USA; ^7^ Preston Robert Tisch Brain Tumor Center, Duke University Medical Center, Durham, NC, USA; ^8^ Department of Neurological Surgery, Washington University School of Medicine, St. Louis, MO, USA; ^9^ Center for Human Immunology and Immunotherapy Programs, Washington University School of Medicine, St. Louis, MO, USA

**Keywords:** pituitary adenoma, PD-L1, RNAscope, checkpoint inhibition, immunotherapy

## Abstract

**Purpose:**

Subsets of pituitary tumors exhibit an aggressive clinical courses and recur despite surgery, radiation, and chemotherapy. Because modulation of the immune response through inhibition of T-cell checkpoints has led to durable clinical responses in multiple malignancies, we explored whether pituitary adenomas express immune-related biomarkers that could suggest suitability for immunotherapy. Specifically, programmed death ligand 1 (PD-L1) has emerged as a potential biomarker whose expression may portend more favorable responses to immune checkpoint blockade therapies. We thus investigated the expression of PD-L1 in pituitary adenomas.

**Methods:**

PD-L1 RNA and protein expression were evaluated in 48 pituitary tumors, including functioning and non-functioning adenomas as well as atypical and recurrent tumors. Tumor infiltrating lymphocyte populations were also assessed by immunohistochemistry.

**Results:**

Pituitary tumors express variable levels of PD-L1 transcript and protein. PD-L1 RNA and protein expression were significantly increased in functioning (growth hormone and prolactin-expressing) pituitary adenomas compared to non-functioning (null cell and silent gonadotroph) adenomas. Moreover, primary pituitary adenomas harbored higher levels of PD-L1 mRNA compared to recurrent tumors. Tumor infiltrating lymphocytes were observed in all pituitary tumors and were positively correlated with increased PD-L1 expression, particularly in the functional subtypes.

**Conclusions:**

Human pituitary adenomas harbor PD-L1 across subtypes, with significantly higher expression in functioning adenomas compared to non-functioning adenomas. This expression is accompanied by the presence of tumor infiltrating lymphocytes. These findings suggest the existence of an immune response to pituitary tumors and raise the possibility of considering checkpoint blockade immunotherapy in cases refractory to conventional management.

## INTRODUCTION

Pituitary tumors are the second most common intracranial neoplasms, comprising 10-15% of diagnosed brain tumors [1, 2]. Some pituitary tumors are considered “functioning” and liberate physiologic hormones to a pathologic degree to manifest as one of several classic endocrinologic syndromes. Specifically, tumors producing growth hormone (GH) or adrenocorticotropic hormone (ACTH) are associated with significant systemic medical morbidity. In contrast, other pituitary tumors are hormonally silent (non-functioning) but may compress adjacent neurovascular structures during growth, conferring degrees of visual loss, pituitary dysfunction, and cranial neuropathies. Although often cured by surgery alone or controlled medically, as in the case of most prolactin-secreting tumors, recurrence rates are not insignificant. In a large cohort of patients followed for 10 years after transsphenoidal surgery, rates of recurrence across tumor subtypes varied between 6-25% [3]. In the recurrent setting, adjuvant medical treatments in functioning and non-functioning tumors are only variably effective in durable tumor control, and radiotherapy may be limited by proximity to the optic nerves. Other subclasses of pituitary tumors that may be difficult to manage include atypical adenomas and those that invade the cavernous sinus and surround one or both cavernous carotid arteries. Overall, there are a limited number of adjuvant treatment options for clinically challenging pituitary tumors.

The use of immune-based therapies has led to durable clinical responses in patients with a range of systemic cancers [4-7]. In particular, treatments that block T cell “checkpoints”—such as the negative regulator proteins cytotoxic T-lymphocyte-associated protein 4 (CTLA-4) and programmed cell death 1 (PD-1)/programmed death ligand-1 (PD-L1)—unleash tumor-specific immune responses and are now FDA-approved to treat melanoma, lung cancer, and renal cell carcinoma. PD-1 is inducible on tumor infiltrating lymphocytes [8-11], while one of its ligands, PD-L1, may be overexpressed by tumor cells or antigen-presenting cells. The binding of PD-L1 to PD1 represents an “immune checkpoint” in that it impairs the function of activated lymphocytes in peripheral tissues [12-16], providing a potential mechanism by which tumors may evade the immune response [14, 17, 18]. Importantly, in several cancers, PD-L1 expression is positively correlated with improved responses to anti-PD-1/PD-L1 blockade [5, 18], though immunotherapy has also been shown to benefit of a subset of patients whose tumors do not express PD-L1 [19, 20]. Recently, PD-L1 was found to be highly expressed in both glioblastoma [21, 22] as well as high-grade meningioma [23], raising the possibility of checkpoint inhibition in central nervous system tumors, for which clinical trials are ongoing.

Thus, due to the dearth of adjuvant therapies for pituitary tumors refractory to conventional treatments and the recent success of checkpoint blockade immunotherapies in cancer, we investigated the expression of PD-L1 in a range of human pituitary tumors. We determined the level of expression first by RNAscope analysis, and then confirmed PD-L1 protein expression by immunohistochemistry. Lastly, we characterized the immune cell infiltrates in these tumors.

## RESULTS

### Pituitary adenomas express variable levels of PD-L1

**Figure 1:**
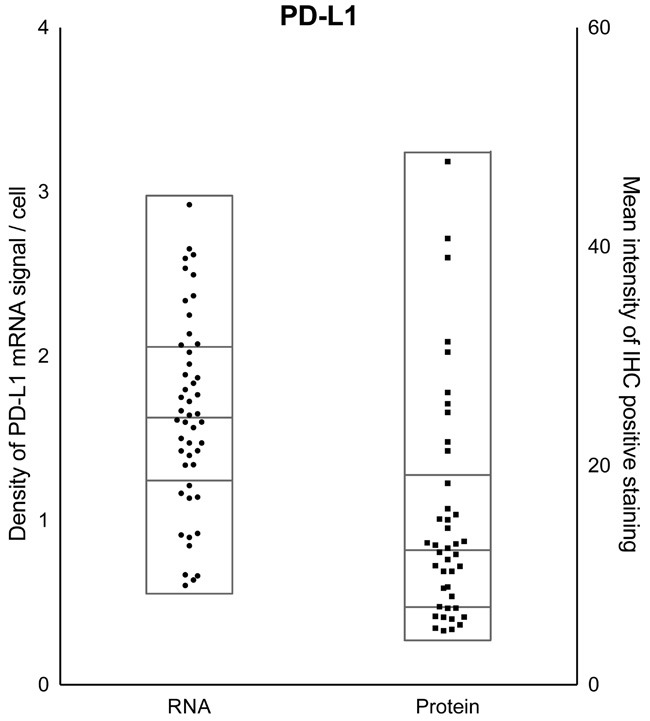
PD-L1 expression across pituitary adenomas Quantification of PD-L1 mRNA (left y-axis) and protein (right y-axis) levels across all pituitary tumors in the cohort reveals diversity in expression. Samples within each scatter plot are divided into quartiles, centered around the median.

We first assessed PD-L1 expression profiles in pituitary tumors by investigating mRNA and protein levels across pituitary adenomas. We used RNAscope *in situ* hybridization to detect PD-L1 mRNA and IHC to assess PD-L1 protein expression. Across all pituitary adenomas, variable expression of PD-L1 was observed (Figure 1), with a 10-fold difference in protein levels and a 5-fold difference across mRNA signals.

### PD-L1 mRNA and protein levels are increased in functioning adenomas compared to non-functioning adenomas

**Figure 2:**
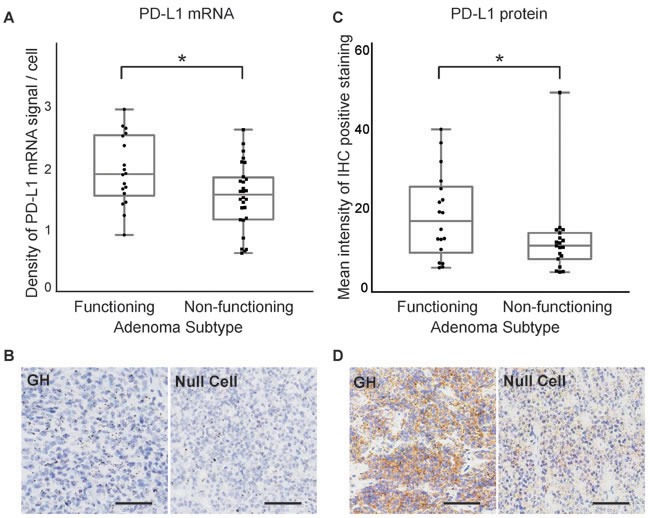
PD-L1 mRNA and protein expression in functioning and non-functioning pituitary tumors **A.** Quantification of PD-L1 RNAscope staining in functioning (*n* = 28) and non-functioning (*n* = 20) pituitary adenomas. **B.** *In situ* hybridization of PD-L1 mRNA using a PD-L1-specific RNAscope probe in a functioning (GH) and a non-functioning (null cell) pituitary tumor. **C.** Quantification of PD-L1 IHC staining in functioning and non-functioning pituitary adenomas. **D.** IHC staining of PD-L1 protein in a functioning (GH) and a non-functioning (null cell) pituitary adenoma. *p < 0.05, scale bar 50μm.

We next explored the relationship between PD-L1 expression and subclasses of pituitary adenomas. Comparison of functioning (GH and PRL expressing, *n* = 28) and non-functioning (null cell and silent gonadotroph, *n* = 20) adenomas revealed significantly higher mRNA levels in the functioning tumors (*p* = 0.023, Figure 2A-2B). Consistent with PD-L1 transcript levels, functioning adenomas harbored statistically higher PD-L1 protein expression compared to non-functioning adenomas (*p* = 0.039, Figure 2C-2D). However, little correlation was observed between PD-L1 mRNA and protein levels at an individual sample level (Supplementary Figure 1). Somatotroph adenomas and mammosomatotroph adenomas did not vary significantly in their PD-L1 expression.

### PD-L1 is expressed in recurrent and atypical adenomas

**Figure 3:**
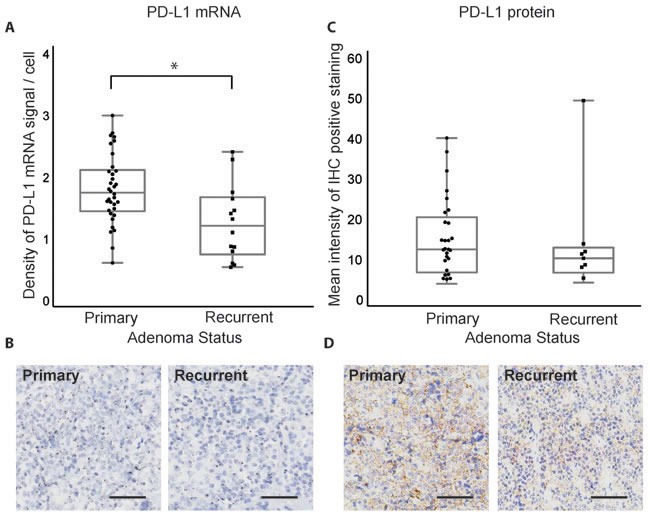
PD-L1 mRNA and protein expression in primary and recurrent pituitary tumors **A.** Quantification of PD-L1 RNAscope staining in primary (*n* = 34) and recurrent (*n* = 14) pituitary adenomas. **B.** RNAscope staining of PD-L1 mRNA in a primary and a recurrent pituitary tumor. **C.** Quantification of PD-L1 IHC staining in primary and recurrent pituitary adenomas. **D.** IHC staining of PD-L1 protein in a primary and a recurrent pituitary adenoma. *p < 0.05, scale bar 50 μm.

**Figure 4:**
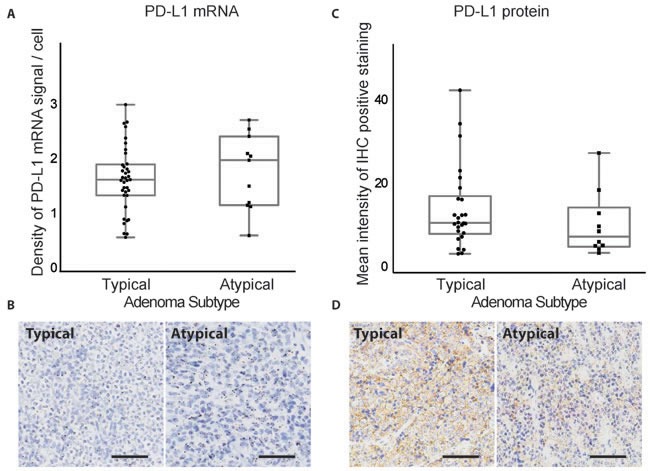
PD-L1 mRNA and protein expression in typical and atypical pituitary tumors **A.** Quantification of PD-L1 RNAscope staining in typical (*n* = 37) and atypical (*n* = 11) pituitary adenomas. **B.** RNAscope staining of PD-L1 mRNA in a typical and an atypical pituitary tumor. **C.** Quantification of PD-L1 IHC staining in typical and atypical pituitary adenomas. **D.** IHC staining of PD-L1 protein in a typical and an atypical pituitary tumor. Scale bar 50μm.

We next investigated PD-L1 levels in recurrent and atypical pituitary adenomas, classes of tumors that can be challenging to manage with existing treatment strategies. PD-L1 transcript, but not protein levels, was significantly elevated in primary (*n* = 34) compared to recurrent (*n* = 14) pituitary adenomas (*p* = 0.0048 for transcript, *p* = 0.316 for protein, Figure 3). Atypical tumors were classified by WHO criteria, as adenomas with excessive p53 immunoreactivity or increased mitotic activity [1]. While PD-L1 mRNA and protein are expressed, they did not vary significantly between adenomas with typical (*n* = 37) or atypical (*n* = 11) status (*p* = 0.54 and 0.21, respectively, Figure 4).

**Figure 5:**
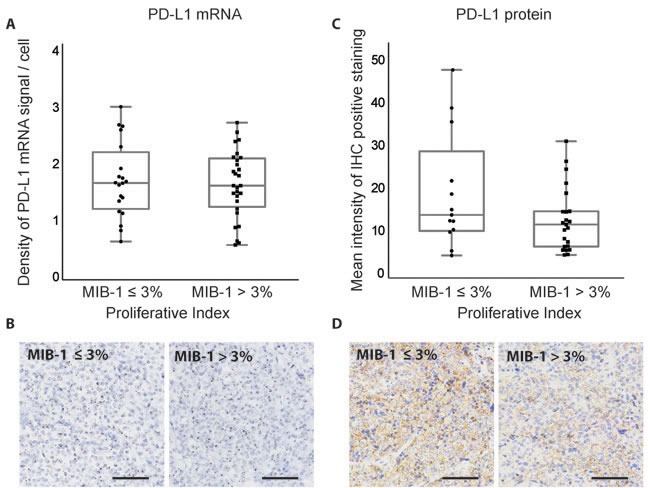
Correlation between MIB-1 proliferative index and PD-L1 expression in pituitary adenomas **A.** Quantification of PD-L1 RNAscope staining in pituitary adenomas with different proliferation indices. **B.** RNAscope staining of PD-L1 mRNA in pituitary adenomas with different proliferation indices. **C.** Quantification of PD-L1 IHC staining in pituitary adenomas with different proliferation indices. **D.** IHC staining of PD-L1 protein in pituitary adenomas with different proliferation indices. Scale bar 50μm.

The MIB-1 proliferative index has been shown to independently predict tumor recurrence or invasiveness and is elevated in atypical adenomas [26, 27]. We further examined the expression of PD-L1 in adenomas with MIB-1 ≤ 3% (*n* = 20) or MIB-1 > 3% (*n* = 28). No significant correlation was observed between PD-L1 mRNA or protein expression and MIB-1 index (Figure 5).

### Characterization of the immune infiltrate of pituitary tumors in tissue specimens

**Figure 6:**
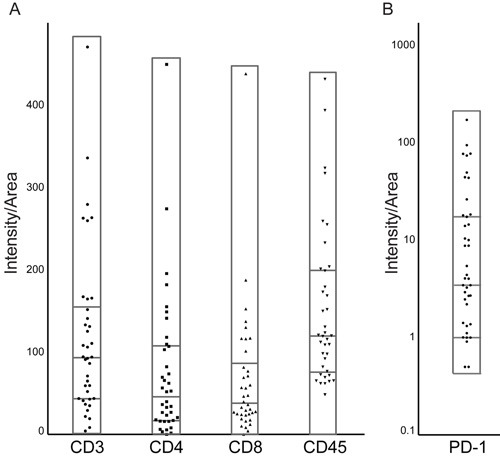
Immune infiltrate markers and PD-1 expression across pituitary adenomas Pituitary tumors express variable levels of **A.** lymphocytic markers, including the T-lymphocyte markers CD3, CD4, and CD8, as well as the pan-lymphocyte marker CD45, and **B.** PD-1. Protein expression is normalized to intensity/cell surface area. Samples within each scatter plot are divided into quartiles, centered around the median.

Having characterized PD-L1 expression in our cohort of pituitary tumors, we investigated the presence of lymphocytic infiltrate in these specimens. We stained TMAs with antibodies targeting a pan-lymphocyte marker (CD45), T cell markers (CD3, CD4, CD8) and the immune regulatory receptor PD-1. An analysis of all tumors taken together shows variable CD3, CD4, CD8, CD45, and PD-1 expression (Figure 6).

**Figure 7:**
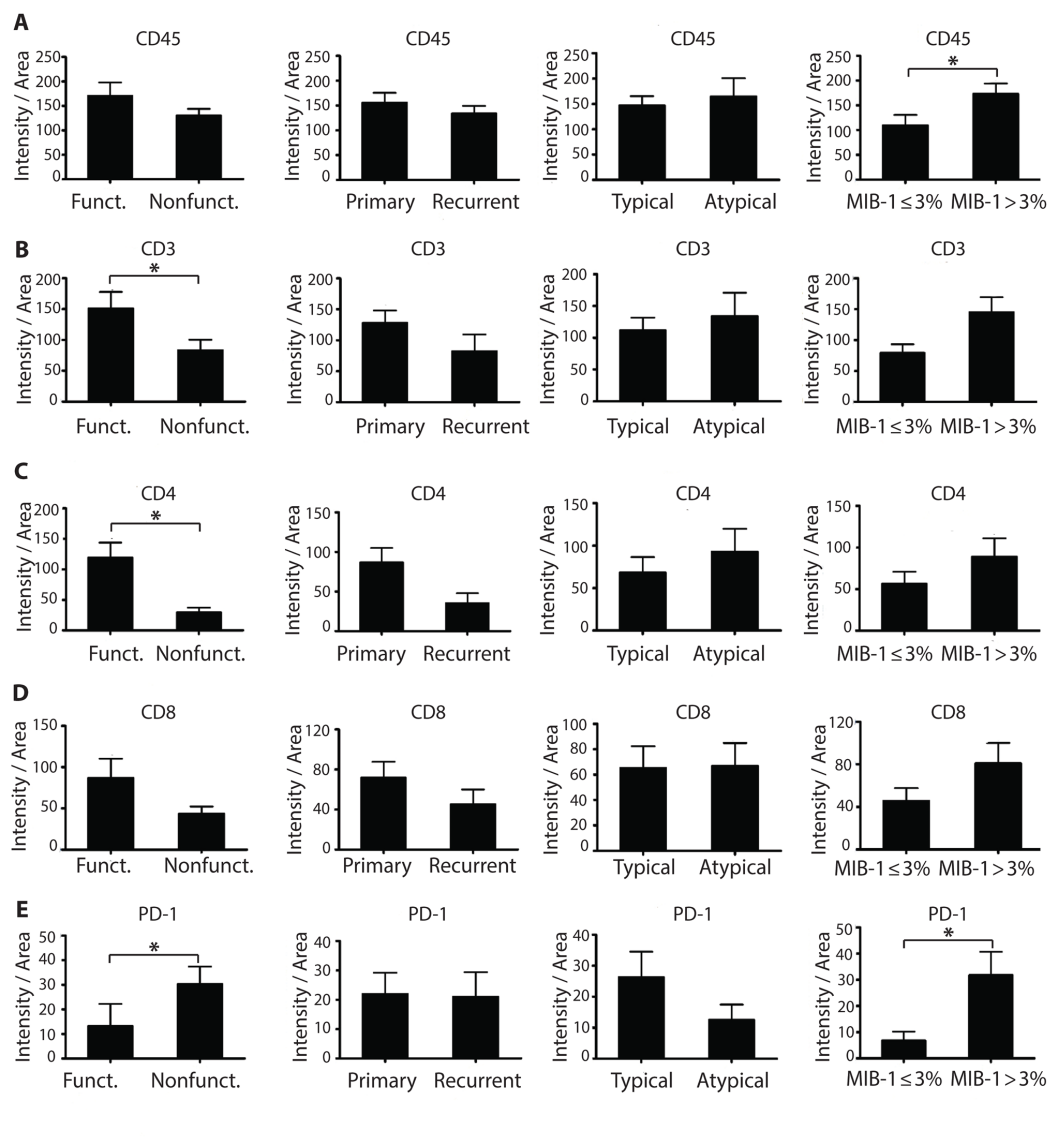
Characterization of the immune infiltrate of pituitary tumors in tissue specimens **A.** Pan-lymphocyte marker CD45 expression in pituitary tumors classified according to tumor function, recurrence status, aggressiveness level, or proliferation status. **B.** CD3+ T lymphocyte, **C.** CD4+ T lymphocyte, **D.** CD8+ T lymphocyte and **E.** PD-1 expression in pituitary tumors. *p < 0.05.

When analyzed by subclass of tumor (Figure 7), both CD3+ and CD4+ populations were significantly increased in functioning adenomas compared to non-functioning adenomas (*p* = 0.019 and *p* = 0.0001, respectively). All lymphocytic infiltrate markers appeared higher in adenomas with an elevated proliferative index, with a significantly increased expression in CD45+ cell infiltrates in adenomas with high (MIB-1 > 3%) compared to low (MIB-1 ≤ 3%) proliferative indices (*p* = 0.0093). Furthermore, CD3+, CD4+, and CD45+ cells trended to a higher expression in atypical adenomas compared to typical adenomas. PD-1 was significantly increased in non-functioning adenomas (*p* = 0.0018) and those with elevated proliferative indices (*p* = 0.003), while being comparable in primary and recurrent tumors.

## DISCUSSION

Substantial work over the last fifteen years has begun to clarify the complex relationship between the immune system and cancer. Specifically, the “cancer immunoediting hypothesis” articulates that tumors dynamically evolve to evade immune responses in order to grow progressively [28]. This shared cancer phenotype of immunoevasion is recognized as a “hallmark of cancer” [29]. Capitalization on the molecular basis of immune regulation has led to the application of checkpoint blockade immunotherapy in several tumor types, with durable clinical responses in some patients with advanced metastatic cancer.

We investigated several immunological parameters in pituitary tumors with the goal of exploring new therapeutic avenues for treatment-refractory tumors. In particular, the location of the pituitary gland outside the blood brain barrier may render systemic therapies more effective. Using *in situ* RNA hybridization and immunohistochemistry, we assessed the expression of the immunoregulatory checkpoint molecule PD-L1 as well as the infiltrating lymphocyte population within a broad range of pituitary tumors. Together, our findings are the first to demonstrate expression of both PD-L1 mRNA and protein in pituitary tumors irrespective of tumor hormone secretion, proliferative index, aggressiveness level, or recurrence status. Previous studies have reported the presence of T-cell enriched populations of tumor infiltrating lymphocytes in pituitary adenomas [30, 31]. Functional subtypes (e.g. GH adenomas) appear to demonstrate greater degrees of lymphocyte infiltration than non-functional tumors [32]. In our study, functional pituitary tumors exhibited more frequent lymphocytic infiltration than non-functional tumors, consistent with previous observations [31]. Ongoing work is directed at expanding our analysis in order to increase our statistical power to clarify the tumor-to-tumor differences in immune cell subsets and cell surface activation markers.

The interaction between PD-L1 and the PD-1 receptor on T cells leads to an inhibitory signal that constrains the function of activated lymphocytes [33]. Thus, PD-L1 overexpression is postulated to be a strategy employed by tumors to evade anti-tumor immune responses. Several mechanisms underlying PD-L1 upregulation have been described that can broadly be considered either “intrinsic” or “extrinsic” in nature. Specifically, increased PD-L1 expression appears to be driven by a number of cell-intrinsic programs—in the setting of *PTEN* loss in malignant brain tumors [21], activating *EGFR* mutations in non-small cell lung cancer [34], and increased STAT3 and AP-1 transcriptional activation in *BRAF-*mutant melanomas resistant to BRAF inhibition [35]. Alternatively, cytokines produced in the tumor microenvironment may also drive PD-L1 expression in an “extrinsic” manner. For instance, interferon-gamma secretion by lymphocytes leads to target cell PD-L1 upregulation. This finding stimulated the concept of “adaptive immune resistance” whereby tumor cells responding to anti-tumor lymphocytes may increase the expression of inhibitory molecules such as PD-L1 [16]. Concomitant presence of lymphocyte populations and PD-L1 expressing cells in pituitary adenomas, as shown in this study, suggests that pituitary tumors might invoke such an adaptive immune resistance mechanism, although the possibility of concomitant intrinsic programs remain. Further work will be needed to determine whether these, or other novel, regulatory pathways converge on PD-L1 overexpression in pituitary tumors.

The selective increase in PD-L1 expression observed in functional pituitary tumors raises a number of future areas of investigation. Our group has characterized the genomic landscape of a broad range of pituitary adenomas including functional and non-functional tumors, and there do not appear to be recurrent alterations in some of the genes, such as *PTEN* and *EGFR*, that have been implicated in PD-L1 upregulation in other tumor types. However, we observed significant differences in broad copy number alterations in the functional subset compared to other pituitary tumors. Ongoing work is directed at exploring whether (a) other shared signaling pathway components downstream of PTEN and EGFR alterations may be more activated in functional tumors; (b) there are recurrent copy number alterations in pathway components that may be relevant to PD-L1 expression or regulation; and (c) a globally altered copy number state itself may be an activator of PD-L1 upregulation.

The clinical success of PD-1/PD-L1 blockade has generated enthusiasm in exploring the spectrum of tumor types that may respond to checkpoint inhibition. However, although durable responses to PD-1/PD-L1 therapies have been observed in a range of cancers including melanoma, non-small cell lung cancer, and renal cell carcinoma [36], reliable biomarkers to predict who will respond to immune blockade are still lacking. Presently, substantial work has centered on the use of PD-L1 as a predictive biomarker [37], which motivated our analysis of its expression in pituitary tumors. Initial studies showed patients whose tumors exhibited PD-L1 expression demonstrated improved clinical responses compared to patients with PD-L1 negative tumors when treated with the ant-PD-1 drug, nivolumab [5] or pembrolizumab [38]. Moreover, a recent study in which patients with urothelial cancer were treated with the anti-PD-L1 agent, atezolizumab, showed that PD-L1 expression on tumor immune infiltrate was associated with improved clinical responses [6]. Intriguingly, both high-grade meningioma and high-grade glioma show increased rates of PD-L1 expression, suggesting that PD-L1 may correlate both with tumor aggressiveness and potential response to therapy [39, 40]. However, additional studies have shown that patients with PD-L1 negative tumors may also exhibit clinical responses to anti-PD-1 therapies [41, 42]. Recent work has highlighted the complex regulation of PD-L1 levels, with post-translational modifications playing a substantial role in modulating protein levels [43]. Furthermore, there appears to be large assay-to-assay variability in the assessment of PD-L1 levels, complicating efforts to use expression as a pre-requisite for trial enrollment [20]. Our current understanding of the relationship between observed PD-L1 levels and response to immunotherapy is incomplete, and this remains an active area of investigation across a variety of disciplines. Of note, immunotherapy has been associated with treatment-related hypophysitis, which may be especially relevant in patients with pituitary tumors who are already vulnerable to tumor-related pituitary dysfunction [44].

Other groups have attempted to stratify tumors by the presence of tumor infiltrating lymphocytes and PD-L1 and have used these parameters to create 4 tumor types—i.e, TIL^+^PD-L1^+^, TIL^−^PD-L1^+^, TIL^+^PD-L1^−^, and TIL^−^PD-L1^−^ [45, 46]. There is growing support that “TIL^+^PD-L1^+^” tumors may have the best chance to respond to checkpoint immunotherapy. Thus, in our dataset, it is possible that functional and primary adenomas—in which there is high PD-L1 as well as increased T cells—may be particularly responsive to anti- PD-L1 therapy. In contrast, slowly proliferative pituitary tumors—in which there are decreased T cells that do not express PD-1, lower PD-L1 expression, and may be anergic or hypoactive—may be less responsive. In other CNS tumors, the presence of tumor infiltrating lymphocytes (TILs) has been correlated with improved survival in several malignancies, including gliomas, and is being examined as a potential prognostic biomarker. We anticipate that intracranial tumors may actually harbor distinct immunologic microenvironments depending on location—e.g., extradural or intradural, proximity to commissural organs, and such—and therefore additional work is needed to understand how distinct intracranial compartments differ. Ultimately, although tumor cell PD-L1 expression may predict clinical response to anti-PD-1/PD-L1 therapy, it is clear that additional work is needed to clarify the full scope of its predictive power as well as the contributions of other *in situ* parameters.

## MATERIALS AND METHODS

### Sample selection and preparation

Formalin-fixed, paraffin-embedded human pituitary tumor specimens were collected from the Department of Pathology, Brigham and Women's Hospital, with corresponding clinical records and pathology reports. Hematoxylin and eosin stained sections corresponding to each tumor were reviewed by two neuropathologists (MA, SS) for selection of specimens with greater than 70% estimated tumor purity. 48 tumors (with duplicate cores for most specimens, spanning 94 samples total) were compiled in tissue microarray (TMA) format for subsequent analysis. The study was approved by the Institutional Review Boards of Brigham and Women's Hospital and Dana Farber Cancer Institute, Harvard Medical School.

Growth hormone (GH) and prolactin (PRL) secreting tumors were classified as functioning. Only two ACTH-expressing adenomas were in the cohort and were excluded from analysis. Null cell adenomas and silent gonadotroph adenomas were classified as nonfunctioning.

### RNAscope *in situ* hybridization

PD-L1 transcript levels were detected using RNAscope 2.0 HD brown detection kit (Advanced Cell Diagnostics, Hayward, CA). A custom-designed RNAscope probe (courtesy of Santagata laboratory) was used to stain the pituitary adenoma TMA. As described previously [23], 5 μm paraffin-embedded TMA sections were baked and deparaffinized, and then boiled with pretreatment reagent for 15 minutes. Protease digestion was performed at 40°C for 30 minutes, followed by hybridization for 2 hours at 40°C with Probe-Hs-PDL1-v2 (Advanced Cell Diagnostics, Hayward, CA). The signal was visualized with 3,3′-Diaminobenzidine (DAB) and cell nuclei were counterstained with hematoxylin. Probe-DapB (Advanced Cell Diagnostics, Hayward, CA) and Probe-Hs-PPIB (Advanced Cell Diagnostics, Hayward, CA) were used as negative and positive control, respectively. All slides were digitally scanned using Carl Zeiss Microimaging (Jena, Germany).

### RNAscope analysis

RNAscope staining was analyzed using CellProfiler image analysis software (http://www.cellprofiler.org/) [24]. The pipeline was previously optimized for meningioma TMAs (http://cellprofiler.org/examples/published_pipelines.html) [23]. Briefly, the image was split into hematoxylin and DAB immunopositive layers with the “Unmixcolors” module. Then, the background was removed and the DAB staining spots were highlighted with the “EnhanceOrSuppressFeatures” module. Finally, the relation of spots with their host cells was identified with the “RelateObjects” module. Optimization for the scoring of pituitary tumors was accomplished by adjustment of the threshold for detecting nuclei and *in situ* hybridization signals (dots). The average number of dots per cell in each pituitary case was compared across groups. False positive recognition of stromal cells such as blood cells in regions of hemorrhage as well as dark staining of some nuclei by the counterstain were excluded by visual review. Cases with staining artifacts or poor tissue integrity following hybridization processing were excluded.

### Immunohistochemistry

Pituitary TMA slides were baked at 60°C for one hour to melt excess paraffin. Immunohistochemistry (IHC) staining for CD3 (1:250, Dako, Carpinteria, CA), CD4 (1:80, Dako, Carpinteria, CA), CD8 (1:100, Dako, Carpinteria, CA), CD45 (1:50, Dako, Carpinteria, CA), PD-L1 (courtesy of Gordon Freeman and validated previously [23], 1:125, diluted in Ventana diluent) and PD-1 (courtesy of Gordon Freeman, 1:1000, diluted in Bond diluent) was performed on a Bond III automated IHC stainer using the Bond Refine Detection Kit (Leica Biosystems, Buffalo Grove, IL). Antigen retrieval was performed using Bond Epitope Retrieval 2 system for 30 minutes. Tissue stained for PD-L1 used an optimized protocol for PD-L1 that consisted of two hour primary antibody incubation, while tissue stained for PD-1 and CDs utilized an optimized protocol that consisted of a 30 min primary antibody incubation. Slides were dehydrated in 85-100% ethanol, mounted using xylene, and cover-slipped. All slides were digitally scanned using Carl Zeiss Microimaging (Jena, Germany).

Murine B cells transfected with human PD-L1 gene served as a positive control for PD-L1 IHC antibody testing (Supplementary Figure 2A-2B). Human tonsil tissue are known to harbor robust PD-1 protein expression within germinal centers, but not outside of them, and served as the control tissue for PD-1 IHC antibody testing (Supplementary Figure 2C).

### Immunohistochemical quantification

Histochemical staining was quantified by color deconvolution as previously described [25]. The TMA IHC images were split into single case images and analyzed using NIH Image J software (http://imagej.nih.gov/ij/). Single DAB stained images were obtained using color deconvolution. After adjustment of the color threshold, the intensity of the DAB positive staining was measured. A mean value (intensity/area) was used for statistical analysis. PD-L1 IHC staining intensity was also measured using color deconvolution and quantified by Image J. PD-1 and CDs IHC staining were quantified by blinded counting. Cases with staining artifacts or poor tissue integrity following immunohistochemical processing were excluded.

### Statistical analysis

Results are expressed as median ± interquartile range, within minimum to maximal values. Statistical analysis was performed using GraphPad Prism (GraphPad, La Jolla, CA), and comparisons were made using unpaired Mann-Whitney test. A *p* value < 0.05 was considered significant.

## SUPPLEMENTARY MATERIALS FIGURES



## Abbreviations

ACTH: adrenocorticotropic hormone
CTLA-4: cytotoxic T-lymphocyte-associated protein 4
DAB, 3: 3′-Diaminobenzidine;
FSH: follicle-stimulating hormone
GH: growth hormone
IFNγIHC: immunohistochemistry
LH: luteinizing hormone
PD-1: programmed cell death-1
PD-L1: programmed death-ligand 1
PRL: prolactin
TIL: tumor infiltrating lymphocyte
TMA: tissue microarray
TSH: thyroid-stimulating hormone
