# Supplementary material for: Increased expression of programmed death ligand 1 (PD-L1) in human pituitary tumors
Source: Oncotarget. 2016 Sep 17;7(47):76565–76. doi: 10.18632/oncotarget.12088 (PMC5363530; doi:10.18632/oncotarget.12088)
Supplement: Supplementary file 1 [file oncotarget-07-76565-s001.pdf]

Increased expression of programmed death ligand 1 (PD-L1) in human pituitary tumors

Supplementary Material

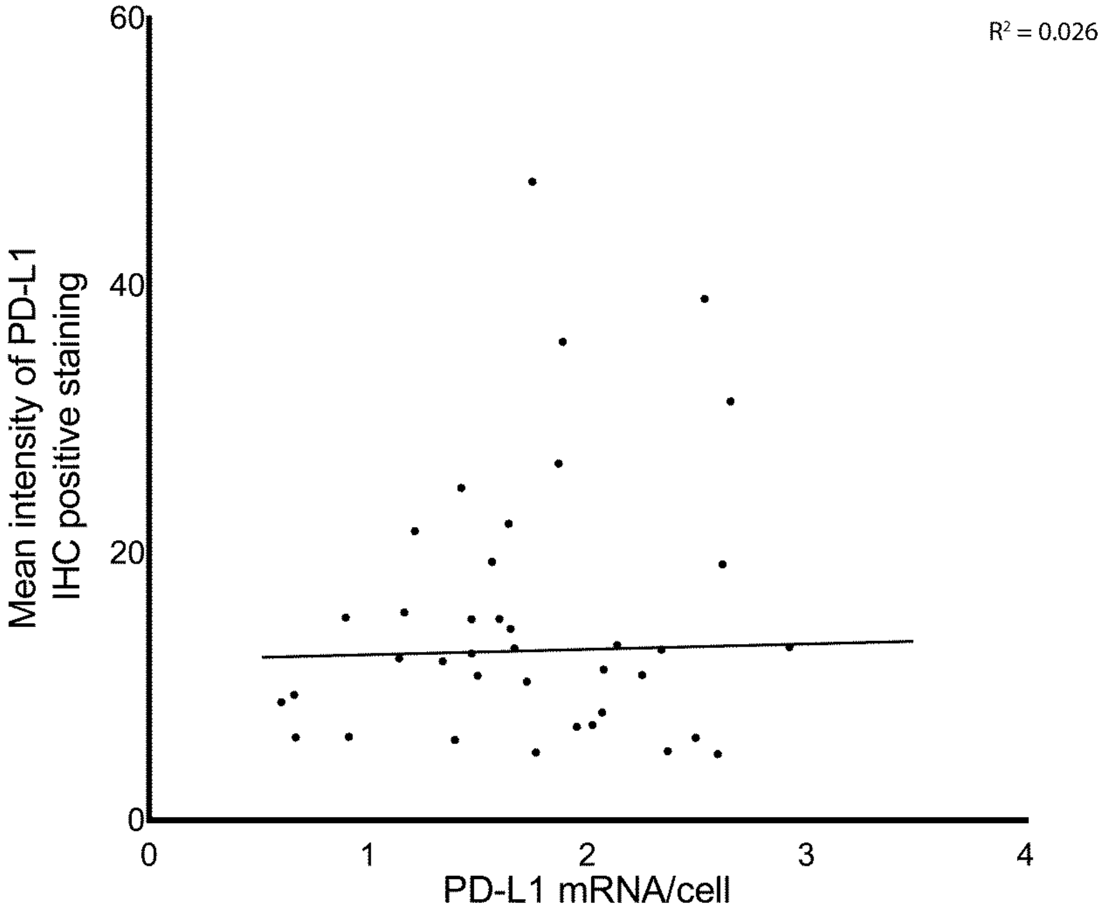

Supplementary Figure 1. Comparison of PD-L1 mRNA and protein expression across pituitary adenomas. Sample level PD-L1 mRNA levels (x-axis) did not correlate with protein levels (y-axis) in this study cohort.

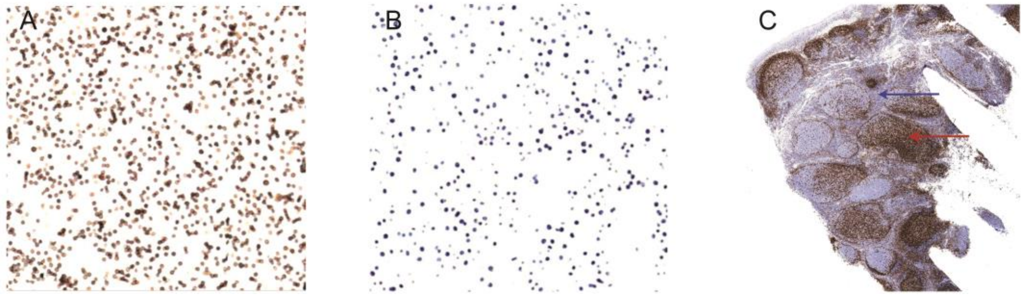

Supplementary Figure 2. **PD-L1 and PD-1 immunohistochemistry staining controls.** Murine B cells (A) with and (B) without transfection of the human PD-L1 gene were stained with the PD-L1 antibody and served as a positive and negative control, respectively, for immunohistochemistry (4X magnification;

DAB staining for PD-L1 with hematoxylin counterstain for background cells). (C) Human tonsil tissue was stained for PD-1 protein, with expected positive expression (red arrow) in germinal centers and negative expression outside of the germinal center (blue arrow).
